# Elevated NLR May Be a Feature of Pediatric Brain Cancer Patients

**DOI:** 10.3389/fonc.2019.00327

**Published:** 2019-04-30

**Authors:** Michal Yalon, Amos Toren, Dina Jabarin, Edna Fadida, Shlomi Constantini, Ruty Mehrian-Shai

**Affiliations:** ^1^Pediatric Hemato-Oncology, Edmond and Lilly Safra Children's Hospital and Cancer Research Center, Sheba Medical Center, Ramat Gan, Israel; ^2^The Sackler School of Medicine, Tel-Aviv University, Tel Aviv, Israel; ^3^Department of Pediatric Neurosurgery, Dana Children's Hospital, Tel-Aviv-Sourasky Medical Center, Tel Aviv, Israel

**Keywords:** pediatric brain cancer, neutrophils, lymphocytes, NLR, LMR

## Abstract

Pediatric brain tumors are the most common solid tumor type and the leading cause of cancer-related death in children. The immune system plays an important role in cancer pathogenesis and in the response to immunotherapy treatments. T lymphocytes are key elements for the response of the immune system to cancer cells and have been associated with prognosis of different cancers. Neutrophils on the other hand, which secrete pro-angiogenic and anti-apoptotic factors, enhance the ability of tumor cells to grow and develop into metastases. We conducted a retrospective study of 120 pediatric brain cancer patients and 171 elective pediatric patients hospitalized in Dana Children's Hospital and Sheba Medical Center. Data on age, sex, treatment, lymphocyte, neutrophil, and monocyte count were collected from routinely performed preoperative blood tests. Neutrophil-to-lymphocyte ratio (NLR), and the lymphocyte-to-monocyte ratio (LMR) were calculated and significance was determined by paired *T* test. *p* < 0.05 was considered as statistically significant. NLR was significantly higher in the pediatric brain cancer patients. The high NLR in pediatric brain cancer patients is the result of a combination of low lymphocytes and high neutrophils. Both of these factors can have a role in cancer development and propagation and also in response to therapy.

## Introduction

Pediatric brain tumors are the most common solid tumor type and the leading cause of cancer-related deaths in children aged 0–14 years (1). There is increasing evidence that malfunction of immune system may contribute to tumor development. Failure to eliminate tumor cells or keeping the tumor cells in a dormant state by the immune system are included in the “hallmarks of cancer” (2). Understanding these underlying mechanisms has provided a basis for the development of new immunotherapies against tumors.

Lymphocytes have, among others, an anti-tumor effect (3). T lymphocytes are key elements to eliminate cancer cells and their dysfunction have been associated with development of cancer (4) and prognosis for several cancer types.

Neutrophils and monocytes on the other hand, may promote tumor progression (5). Neutrophils which secrete pro-angiogenic and anti-apoptotic factors, enhance the ability of tumor cells to grow and develop into metastases (6). Elevated numbers of neutrophils in the peripheral blood is associated with poor outcome in several types of cancers (7). Tumor-associated neutrophils can acquire a pro-tumor phenotype, supporting tumor growth, and suppressing the antitumor immune response (8). Myeloid-derived suppressor cells (MDSC) which are closely related to neutrophils and monocytes (9) play a major role in suppression of T cells (10, 11). Not only are levels of MDSC increased in the blood of patients with cancer, MDSC frequencies correlate to clinical cancer stage in several cancers including bladder cancer (12) and breast cancer (13).

Neutrophils, lymphocytes and monocytes have prognostic value in patients with a variety of common solid tumors (14). These patients are characterized by an increase in circulating neutrophil levels accompanied by a fall in circulating lymphocyte levels thus the neutrophil to lymphocyte ratio (NLR) is increased. In general, the blood NLR is high in patients with more advanced or aggressive cancers (15) and correlates with poor survival of patients with many solid tumors (16, 17).

Lymphocyte-to-monocyte ratio (LMR) has been suggested to be an important factor for predicting prognosis in patients with lung cancer, colon cancer and hematologic malignancies (18, 19). In a meta-analysis of adult solid tumors it was concluded that low pre-treatment LMR is an unfavorable prognostic factor (20).

In this study we investigated NLR and LMR in pediatric brain cancer patients on day of diagnosis before any treatment administration.

## Methods

Clinical data from pediatric brain cancer patients (M = malignant group) and elective pediatric patients hospitalized for hernia repair were retrospectively analyzed. Exclusion criteria were steroid treatment before the blood test and a history of concomitant diseases. Age, Sex, treatment, blood count plus differential were collected from blood tests performed before surgery in both groups. NLR, and the LMR were calculated and significance was determined by paired *T*-test. *p* < 0.05 was considered as statistically significant. ANOVA test was used to compare mean of NLR value in different brain tumor types.

## Results

Clinical data from 120 pediatric brain cancer patients and 171 elective pediatric patients hospitalized for hernia repair were retrospectively analyzed. The study was approved by the ethical review boards of both Sheba and Tel Aviv Sourasky Medical Centers and was consistent with the declaration of Helsinki. Cancer patients who received dexacort before the blood test were excluded. Children from the elective hernia surgery control group with known background diseases were also excluded. Ninety-nine cancer and 62 control patients that met the inclusion criteria and had all the data in the medical records were included in the analysis. Brain cancer patients consisted of 37 astrocytoma patients, 27 medulloblastoma patients, 25 patients with pilocytic astrocytoma, and 10 glioblastoma patients. Age, Sex, treatment, lymphocyte, Neutrophil count, and monocyte count were collected from routinely performed preoperative blood tests. NLR and the LMR were calculated and significance was determined by paired *T*-test. *P* < 0.05 was considered as statistically significant.

Male/female ratios in the study and control group were 64%/36% and 61%/29%, respectively. Mean age was 10.26 and 8.7 years old, respectively. No significant NLR and LMR differences were found between males and females in the patient group. Table 1 summarizes the blood count results, differential, NLR, and LMR in both groups.

**Table 1:** Blood count results, differential, NLR, and LMR in brain cancer and hernia groups.

**Parameter**	**Brain cancer**	**SD**	**Hernia**	**SD**	***p*-value**
White blood cells	10.2 X 109/L	5.3	11.2X109/L	10.9	0.550
Neutrophils	64.3%	16.5	56.1%	16.7	0.001
Lymphocytes	27.1%	14.3	32.0%	14.9	0.009
Monocytes	6.1%	2.9	3.3%	3.3	0.001
NLR	4.5	5.4	2.9	3.4	0.025
LMR	5.3	3.9	4.2	2.5	0.086

*SD, Standard deviation*.

The mean percentage of neutrophils was higher in the patient group (64.3%) compared to the control group (56.11%) *p* = 0.001. In contrast, the mean percentage of lymphocytes was significantly higher in the control group (32.08%) compared to the patient group (27.11%) *p* = 0.009. As a result, the NLR was significantly higher in the patient group (4.59) compared to the control group (2.96) *p* = 0.025. Although the mean percentage of monocytes was higher in the patient group (6.1%) compared to the control group (3.3%) *p* = 0.001, there was no significant difference in LMR between the patient group (5.3) compared to the control group (4.2) (*p* = 0.08).

Table 2 summarizes the mean NLR results in different brain tumor types in children. Although NLR values of astrocytoma and pilocytic astrocytoma are lower than glioblastoma and medulloblastoma, no significant difference was found between the mean of NLR value in different tumor types by ANOVA test {*F* = 3.75, *p* = 0.13}.

**Table 2:** NLR value results in different brain tumor types in children.

**Tumor type**	***N***	**NLR**
Astrocytoma	37	3.7
Medulloblastoma	27	6.0
Pilocytic astrocytoma	25	3.3
Glioblastoma	10	5.4

*N, number of samples*.

## Discussion

Pediatric cancers and especially solid tumors can have devastating effects on young lives and their families. There is increasing evidence that failures of the immune system may contribute to tumor development. Lymphocytes have an important role in tumor cells elimination. Neutrophils and monocytes on the other hand, may promote tumor progression. In fact, suppression of the immune response to cancer cells is one of the impediments to develop effective immunotherapeutic approaches for glioma patients (21). Overcoming the suppression can be done by blocking the inhibitory signals of the immune system by anti CTLA4 and anti PD1/PDL1 therapy (22). On the other hand, persisting response of the inflammatory milieu paves the way to cancer (23). Neutrophils trigger and sustain a state of chronic inflammation (6) and high systematic immune-inflammation is significantly associated with a lower overall survival rate of patients with colon cancer (24), lung cancer (25), and breast cancer (26). Suppressing this arm of the immune response is necessary to limit proliferative signaling, angiogenesis, migration and invasion processes (2). Inhibitors of inflammatory pathways have been successful in pre-clinical tumor models and early clinical trials (27).

It has been shown that elevated NLR is predictive of poorer overall survival in patients with hepatocellular/ squamous cell carcinoma, GBM (28–30), pancreatic ductal adenocarcinoma (31), ovarian cancer (32) and pediatric sarcoma (33). In addition, preoperative elevated NLR reflects a systemic inflammatory response and is a predictor of poor survival in gastric cancer (16). Finally, high NLR is associated with poorer outcomes in patients receiving immune checkpoint inhibitors (34). In our study, pediatric brain cancer patients' blood samples present both increased neutrophil count and decreased lymphocyte count resulting in significantly higher NLR. Moreover, although no significant difference was found between the mean of NLR value in different tumor types in children, NLR values of astrocytoma and pilocytic astrocytoma are lower than glioblastoma and medulloblastoma. It is important to note that pediatric astrocytoma and pilocytic astrocytoma are with a more favorable prognosis compared to glioblastoma and medulloblastoma.

Using the body's own immune system to fight cancer is increasingly desirable treatment for cancer because of its' potential to specifically target the tumor while limiting damage to normal tissue. Because lymphocytes account for only 20–40% of the total WBC count, low levels of T cells may go unnoticed when WBC count is checked without a differential count of CD4+ (helper) T cells/CD8+ (suppressor) T cells/B cells. Reduced lymphocyte numbers may contribute to cancer development and may affect treatment, especially the current promising treatment of immunotherapy. Furthermore, the success of immunotherapy in tumors of the central nervous system depends on the immune microenvironment and lymphocyte tracking through the blood-brain barrier (BBB) into the central nervous system (35). The brain is presumably an immune-privileged organ due to the BBB (36). During gliomagenesis, the BBB is broken and circulating immune cells including T cells, B cells, macrophages, and MDSC cross the BBB (37). The resulting bi-directional communication between immune cells and glioma cells create an immunosuppressed microenvironment that promotes tumor survival and growth (38). Glioma-associated microglia and macrophages are attracted to the tumor (39) and can be polarized into M2 becoming tumor-supportive and immunosuppressive cells (40). The glioma immunosuppressive environment is further enhanced by elevated numbers of regulatory T cells (41). Immunophenotyping of pediatric brain tumors reveal immunosuppressive phenotype in higher grade tumors with more regulatory T cells present in these tumor types (42). Interestingly, it has been shown recently that intracranial tumors like GBM may cause depletion of mature T cells (43). The tumor imposed depletion of Sphingosine-1-phosphate receptor 1 from the T cell surface prevents their trafficking from lymphoid organs into the circulation. It may well be that in pediatric brain cancer patients the T cells are sequestered in the lymph nodes and can inversely be released to fight cancer by manipulations yet to be discovered. Thus, Immunotherapy via adoptive cell transfer, especially with T cells engineered to express chimeric antigen receptors, represents a promising approach (44). Recent evidence has demonstrated that systemic therapy for brain tumors is not limited by the BBB (45). Cellular therapy as well as reversing immune-suppression through immune checkpoint blockade are showing promising results for GBM (46). In light of these findings the prognostic as well as treatment response prediction value of the NLR warrants prospective validation in large cohorts of children with CNS tumors.

## Ethics Statement

The study was retrospective study of clinical files. Clinical data from 120 pediatric brain cancer patients and 171 elective pediatric patients hospitalized for hernia repair were retrospectively analyzed. The study was approved by the ethical review boards of both Sheba and Tel Aviv Sourasky Medical Centers and was consistent with the declaration of Helsinki.

## Author Contributions

RM-S and DJ contributed to implementation, analysis, and interpretation of the data. RM-S, DJ, MY, AT, EF, and SC were involved in experimental design, in the writing of the manuscript, and have read and approved the final version.

### Conflict of Interest Statement

The authors declare that the research was conducted in the absence of any commercial or financial relationships that could be construed as a potential conflict of interest.
